# Unveiling the importance of heterotrophy for coral symbiosis under heat stress

**DOI:** 10.1128/mbio.01966-24

**Published:** 2024-08-29

**Authors:** Stephane Martinez, Renaud Grover, Christine Ferrier-Pagès

**Affiliations:** 1 Centre Scientifique de Monaco, Coral Ecophysiology Team, Monte Carlo, Monaco; 2 Graduate School of Oceanography, University of Rhode Island, Narragansett, Rhode Island, USA; Max Planck Institute for Chemical Ecology, Jena, Germany; King Abdullah University of Science and Technology, Thuwal, Saudi Arabia

**Keywords:** heterotrophy, mixotrophy, CSIA-AA, isotopes, heat stress, bleaching, Symbiodiniaceae, dinoflagellates

## Abstract

Global warming endangers reef-building corals as they lose their photosynthetic symbionts, which limits their ability to feed autotrophically. Consequently, heterotrophy, the capture of zooplankton, can become crucial for the energy budget of heat-stressed corals. However, it is difficult to assess the extent of the heterotrophic contribution in corals, as well as the dynamics of nutrient exchange between the host and its symbionts. In this pioneering study, we employed a suite of isotopic markers, including ^13^C- and ^15^N bulk tissue isotope measurements, compound-specific isotope analysis of amino acids (CSIA-AAs), and ^13^C- and ^15^N-labeled food incubations, to investigate nutrient acquisition and allocation in the coral *Stylophora pistillata* under controlled and heat-induced bleaching conditions. Bulk isotope values and inorganic carbon assimilation remained unchanged in the bleached corals compared to the control corals, overall indicating undisturbed autotrophic activity of the symbionts under heat stress. However, CSIA-AAs showed an increased dependence on heterotrophy for amino acid synthesis in both the host and the symbionts despite reduced assimilation of ^15^N-labeled food. Overall, these results suggest that although *S. pistillata* reduces its assimilation of heterotrophic food under heat stress, the acquisition of amino acids by the coral host and symbionts still relies on heterotrophy. This study emphasizes the importance of using multiple indicators to gain a comprehensive understanding of coral nutrition. It shows that coral dependence on heterotrophy is not only associated with a decline in autotrophic availability. Rather, it demonstrates the ability of *S. pistillata* to adapt its utilization of food sources to the prevailing environmental conditions.

**IMPORTANCE:**

This work highlights that every isotopic marker displays a piece of different information concerning the diet of the model coral *S. pistillata*. By combining all markers, we observed that although *S. pistillata* exhibited reduced heterotrophic assimilation under heat stress, amino acid acquisition and synthesis remained dependent on heterotrophy. The findings emphasize the adaptability of corals in utilizing different food sources, which is vital for their resilience and recovery in changing environmental conditions. This research underscores the complexity of coral symbiosis and highlights the need for multiple indicators to understand dietary dynamics comprehensively.

## INTRODUCTION

Scleractinian corals are the cornerstones of coral reefs, one of the most biodiverse ecosystems on Earth, which provides valuable resources to millions of people living in proximity (1). Corals exhibit high productivity in nutrient-poor waters through efficient nutrient recycling between the coral host, its endosymbiotic Symbiodiniaceae dinoflagellates, and other associated microbes (2, 3). Symbiodiniaceae play a major role in the autotrophic nutrition of corals by supplying their host with carbon-rich molecules, photosynthates, which can cover 90% of the coral’s respiratory requirements under optimal conditions (2, 4). Together with other microbes such as diazotrophs, they can also use inorganic nutrients such as nitrogen and phosphorus dissolved in seawater (5, 6). On the other hand, corals are heterotrophic organisms and can consume a wide range of particles, from bacteria to macrozooplankton. Corals have, therefore, evolved different feeding strategies based on both autotrophic and heterotrophic nutrient acquisition, also known as mixotrophy (7). While some corals are primarily autotrophic, others can switch to a more heterotrophic diet if external food sources are available in their environment (7). Heterotrophy plays a crucial role in maintaining coral metabolism, especially under stressful conditions. Scleractinian corals with higher rates of heterotrophic feeding have higher survival and recovery rates following bleaching than those with limited heterotrophic plasticity (8, 9). This finding has significant implications for the future of corals and coral reefs, which are under threat due to the increasing frequency and severity of marine heat waves (10) that disrupt the symbiotic relationship between corals and Symbiodiniaceae, leading to bleaching and starvation (11, 12).

Although many coral species can be voracious predators under laboratory conditions when given large amounts of prey, the extent to which corals rely on heterotrophy in the wild is poorly understood (13–15). This is due to the wide variety of food sources available, their transformation and recycling within the coral holobiont, the enormous input of autotrophic compounds that often overwhelm any small heterotrophic input, and the lack of suitable methods for tracing individual prey types. In addition, coral predation and assimilation rates can change significantly (increase or decrease) under stress (16), even when corals receive the same quality and quantity of prey, making it even more difficult to understand the extent to which prey supplementation can sustain coral metabolism.

Recently, Compound Specific Isotope Analyses (CSIA) of fatty acids (FAs) and especially amino acids (AAs) have proven useful for identifying the main food sources for cold water corals that are not symbiotic with algae and meet their energy requirements exclusively through heterotrophy (17–20). Amino acids are typically categorized as essential or non-essential, depending on whether consumers can produce them or must obtain them from their food (21). Since essential amino acids can only be synthesized by autotrophs or bacteria, they are passed on to predators with little to no carbon fractionation (21, 22). Their *δ*^13^C values are unique to each group and environment and, therefore, serve as a “fingerprint” for the biosynthetic origin of the carbon. In addition, the *δ*^15^N values of source AAs (e.g., phenylalanine, Phe) remain essentially unchanged (max. 0.5‰) during trophic transfer, while the values of trophic AAs (e.g., glutamic acid, Glu) are enriched (by up to 10‰) with increasing trophic position (23, 24). The *δ*^15^N composition of source vs trophic AAs is informative both for the estimation of the trophic position (TP) and for the *δ*^15^N composition of AAs at the base of the food web. Therefore, the difference between *δ*^15^N -Glu and Phe is used to calculate the trophic position (TP). Corals with a TP of 1 are assumed to rely only on autotrophy, while corals with a TP >1.5 are considered more heterotrophic (25, 26).

In contrast to cold-water corals, the application of *δ*^13^C- CSIA-AAs to symbiotic corals showed either large variability in the values within the same coral population (13) or no significant change between autotrophic and mixotrophic species (25). This suggests that changes in *δ*^13^C-AAs with diet are complex and difficult to interpret, or that the large input of Symbiodiniaceae photosynthates within the symbiotic association may mask the small heterotrophic input from the host. On the contrary, *δ*^15^N CSIA-AAs values of symbionts or coral host tissue can be good indicators of heterotrophy (15, 25–27). Experimental studies have shown that the *δ*^15^N-glutamic acid increases from 3.7‰ in autotrophic corals to 8‰−12.6‰ in mixotrophic and fully heterotrophic corals, respectively (25, 26). In addition, *δ*^15^N-AAs showed a more pronounced change with heterotrophy in Symbiodiniaceae than in coral host tissue, suggesting that symbionts are responding faster to a change in diet (26).

Although some laboratory and *in situ* studies have started to use CSIA-AAs to track coral nutrition, no experiment has examined changes in *δ*^15^N and *δ*^13^C-AAs of corals exposed to thermal stress and bleaching. In such a situation, the use of CSIA-AAs could reveal the ability of corals to transit from an autotrophic to a more heterotrophic diet to compensate for the loss of symbionts and autotrophic food. Therefore, the main objective of this study was to use a suite of isotopic markers, including bulk *δ*^15^N and *δ*^13^C of coral tissue, *δ*^15^N and *δ*^13^C-CSIA-AAs, and ^15^N and ^13^C enrichment of potential food sources, to understand the changes in the diet of the model scleractinian coral, *Stylophora pistillata* during thermal stress. To this end, we experimentally generated two groups of corals maintained at 25°C or 32°C and fed with *Artemia salina* nauplii twice weekly. These experiments were performed to test two hypotheses: (i) *S. pistillata* relies more on heterotrophy when exposed to thermal stress and bleaching; therefore, assimilation of heterotrophic nitrogen, measured with ^15^N-labeled prey, should increase in heat-stressed nubbins, in parallel with a change in bulk *δ*^15^N and *δ*^13^C values of coral tissue, trophic index, and *δ*^15^N/*δ*^13^C-AAs. (ii) All isotopic proxies should converge and show an increase in heterotrophy. The overall goal of this study was to calibrate, under experimental conditions, a set of isotopic markers capable of tracking changes in coral nutrition, with the ultimate goal of using the best markers to track coral heterotrophy in the reef.

## MATERIAL AND METHODS

### Experimental design

The experiments were performed with six colonies of the scleractinian coral *Stylophora pistillata,* which is abundant in the Northern Red Sea. Colonies were grown in controlled conditions in open-water flow aquaria, supplied with oligotrophic seawater pumped from 40 m depth in front of the Oceanographic museum de Monaco, filtered through sand filters, and renewed at a rate of 12 L h^−1^. The temperature was maintained constant at 25°C using heaters connected to Elli-Well PC 902/T controllers. Light (200 µmoles photons m^−2^ s^−1^) was provided with several HQI lamps. Light intensity was controlled by a LI-COR data logger (LI- 1000) connected to a spherical quantum sensor (LI-193). Dozen fragments were produced from each mother colony, and they were allowed to heal for 3–4 weeks. During healing, they were fed twice a week with *A. salina* nauplii at repletion.

Fragments (*n* = 72 in total) were identified and evenly divided into four aquaria (3 fragments/colony/aquaria). Two aquaria were kept at 25°C and two were ramped to 32°C at the rate of 1°C every 3.5 days. During the experiment, corals were fed twice a week with *A. salina* at repletion. Three days after corals reached 32°C, six nubbins (1 per colony, 3 per aquaria) were taken at each temperature for each of the following measurements, described in detail in the following sections: six fragments were sampled for photosynthesis determination and symbionts count. Six fragments were used for natural bulk *δ*^13^C and *δ*^15^N, and CSIA-AA measurements. Six additional fragments were used in a feeding trial with ^15^N-labeled *A. salina*. The remaining 18 fragments were incubated with ^15^N-labeled amino acids, ^15^N-labeled ammonia, or ^13^C-labeled bicarbonate, to determine their assimilation rates of dissolved carbon and nitrogen.

Measurements of net photosynthesis (*P*_n_) and respiration rates (*R*) were performed at 200 µmol photons m^−2^ s^−1^ and in the dark, respectively. Nubbins were individually placed in 50 mL transparent Plexiglas chambers, hermetically sealed, and filled with 0.45 µm filtered seawater (FSW) maintained at 25°C or 32°C. Seawater mixing was ensured with magnetic stirrers. The chambers were equipped with oxygen optodes connected to the Oxy-4 software (Chanel fiber-optic oxygen-meter, Presens, Regensburg, Germany). The optodes were calibrated before each measurement using nitrogen-saturated and air-saturated seawater for 0% and 100% oxygen, respectively. *P*_n_ and *R* were then estimated using a regression of the oxygen data against time, and gross photosynthesis (*P*_g_) was calculated as the sum of the absolute values of *R* and *P*_n_. Samples were then frozen at −20°C for the latter determination of their symbiont density and skeletal surface area. Symbiont density was measured after the tissue was extracted using an air-pick and homogenized using a Potter tissue grinder. Samples were then centrifuged at 500 *g* for 10 min, and symbiont pellets were resuspended in 2 mL. A subsample of 500 µL was used for the determination of symbiont density using a Z1 Coulter Particle Counter (Beckman Coulter). Data were normalized to the surface area (cm^2^) using the wax-dipping technique (28).

For the feeding experiment, ^15^N-labeled *A. salina* nauplii were first prepared according to Tremblay et al. (29). Afterward, each coral nubbin was placed in a 250 mL beaker and fed with one portion of *A. salina* for 5 h (ca. 2,000 *A*. *salina*/mL). After feeding, nubbins were returned to their aquaria for prey digestion, and 3 nubbins from different colonies were sampled after 6 and 72 h. Nubbins were immediately frozen at −80°C until further analysis.

Nubbins were also sampled to assess their uptake rates of labeled amino acids, ammonia, and bicarbonate. Each coral nubbin was placed in a 250-mL beaker on a magnetic stirrer for 2 h. 2 µM ^15^N-labeled-AAs (99% enrichment, Sigma), 2 µM ^15^N-NH4 (99% enrichment, Sigma), or 2 mM ^13^C-bicarbonate (99% enrichment, Sigma) were then added to the beakers, and corals were incubated for 2 more hours. Afterward, nubbins were returned to their aquaria and three nubbins from different colonies and aquaria were sampled after 1 and 72 h. Nubbins were immediately frozen at −80°C until further analysis.

### Sample preparation for isotope and CSIA-AAs analyses

For all nubbins, the tissue was removed from the skeleton using an air-pick with ultra-pure water, and the slurry was homogenized with a potter tissue grinder. The homogenized sample was centrifuged at 500 *g* for 10 min at 4°C to separate host tissue from symbionts. The symbionts were washed twice with ultra-pure water and centrifuged again at 500 *g* for 10 min at 4°C. The two fractions were freeze-dried before subsequent analysis of their CSIA-AAs and bulk stable isotope.

### Bulk isotope analyses from non-labeled and labeled fragments

Approximately 600 µg of lyophilized host and symbiont material was transferred to tin caps to analyze the carbon and nitrogen bulk isotopic values using an Integra II isotope ratio mass spectrometer (Sercon, United Kingdom). Two tin caps were prepared for a separate analysis of the carbon and nitrogen isotopic values, as the material for the carbon isotopic measurements was acidified with a few drops of 10% HCl before the measurements. Results are obtained as *δ*^13^C and *δ*^15^N values or Atom%-^13^C or -^15^N. These Atom% values will be used to calculate C and N assimilation rates, *ρ*_C_ and *ρ*_N_ in ng.cm^−1^, after 5 h, according to the equation of Dugdale and Wilkerson (30):


$$\rho_{X}=\frac{X_{mes}-X_{natural}}{(X_{enr}-X_{mes})\times S}\times M_{sample}\times M_{X}\times 10^{6}$$

Where *X* = C or N,

*X*_mes_: %^13^C or ^15^N measured in the sample,

*X*_natural_: natural abundance in ^13^C or ^15^N in control nubbins,

*X*_enr_: ^13^C or ^15^N enrichment in the incubation medium,

*S*: nubbin surface area in cm^2^,

*M*_sample_: mass of the freeze-dried sample in mg,

*M*_X_: particulate carbon or nitrogen mass (mg) per mg of host tissue or symbiont.

### CSIA-AAs analyses

The nitrogen and carbon isotopic compositions of amino acids were determined by gas chromatography/combustion/isotope ratio mass spectrometry (GC/C/IRMS). The acid-hydrolyzed host and symbiont samples were first derivatized using the Ezfaast kit before isotopic analysis was performed according to Martinez et al. (31). Briefly, 3.5 mg of the sample was acid hydrolyzed using 6 nmol HCl. The hydrolyzed samples were derivatized with the Ezfaast kit with a slight modification of replacing reagent 6 (containing chloroforms that affect the combustion reactor) with dichloromethane as a solvent. Amino acids were separated on a Zebron ZB-50 column (30 m, 0.25 mm, and 0.25 µm) on a Thermo Scientific Trace 1300 Gas Chromatograph using helium as the carrier gas at a constant flow of 1.5 mL/min. For carbon analysis, 1.5 µL was injected in split mode (1:15) at 250°C, while 1.5 µL was injected in splitless mode at 250°C for nitrogen analysis. The separated amino acids were split on a MicroChannel device into two direction flows: Thermo Scientific ISQ quadruples for amino acid identification and Thermo Scientific Delta-V advantage for C and N isotope analysis. In order to determine the isotopic ratio of carbon and nitrogen, the separated amino acids were combusted in a Thermo scientific GC isolink II at 1,000°C for CO_2_ and N_2_. Before the sample was run into the Delta-V for N_2_ analysis, it passed through a cold trap with liquid nitrogen to freeze all other gases. Duplicates for carbon and triplicates for nitrogen were injected from each sample. Stable isotope ratios were expressed in standard *δ* notation, with the standard for carbon being Vienna PeeDee Belemnite (VPDB) and for nitrogen atmospheric N_2_ (air). To account for carbons atoms incorporated during the derivatization process, we followed the correction factor of Docherty et al. (32) for each amino acid. The trophic position (TP_Glu-Phe_) was calculated using glutamic acid and phenylalanine with the predefined equation of Chikaraishi et al. (24) with the constants from Martinez et al. (31).

### Statistics

The statistical analysis of CSIA-AA was performed using R version 4.3.3 with LDM package 6.0.1. To assess the contribution of temperature, host-symbiont fractions, and their interaction to the variation observed in CSIA-AA, a permutational analysis of variance (PERMANOVA) was performed using the “permanovaFL” function from the LDM package. The permanovaFL model included aquaria and colonies as covariates to account for their influence on the results. Subsequently, a PERMANOVA with Monte-Carlo (MC) was performed for pairwise comparisons, using PRIMER-e 7 with the PERMANOVA + add on. The principal component analysis (PCA) was also calculated with PRIMER-e 7. For all other analyses, linear mixed effect models were calculated and tested for significance, using the R package lme4 (version 1.1.35.3) and lmerTest (version 3.1.3) with aquaria and colonies included as random effects to account for their effect. Only values with *P* < 0.05 were considered significant.

## RESULTS

**Fig 1:**
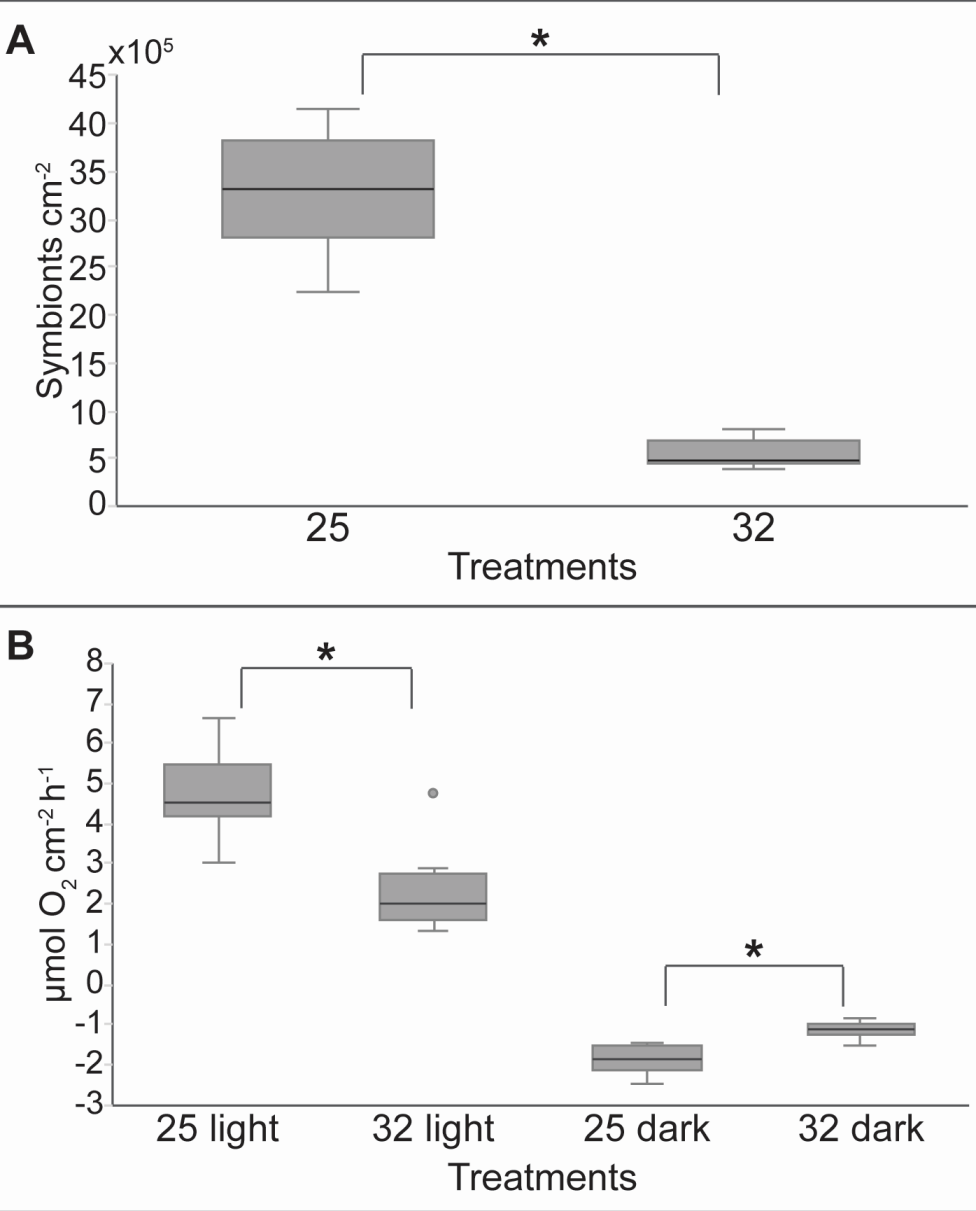
Coral physiology. (A) Number of symbionts (*Symbiodiniaceae*) for 1 cm^2^ of skeletal surface area for the two temperature conditions of 25°C and 32°C. (**B**) Oxygen fluxes at the two temperature conditions of 25°C and 32°C, in the light (photosynthesis) and the dark (respiration). * indicates a significant difference.

**Fig 2:**
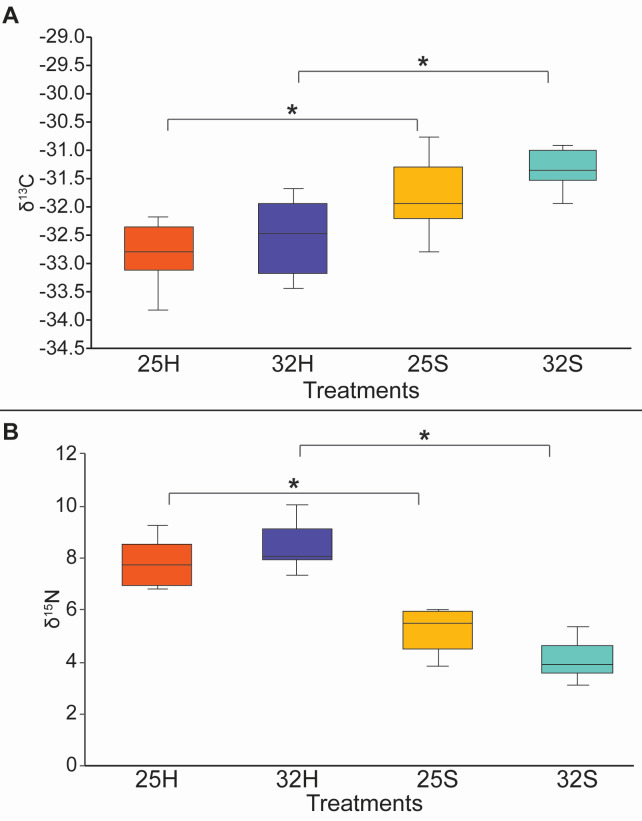
Bulk isotope analysis of the host tissue (**H**) and the symbionts (**S**) of the coral *Stylophora pistillata* at two temperatures of 25°C (25) and 32°C (32). (A) Bulk carbon isotope and (B) bulk nitrogen isotope. * indicates a significant difference.

Symbiont density significantly decreased by more than 80% at 32°C compared to 25°C (Fig. 1A, linear mixed-effects *P* < 0.001). A ca. 50% decrease in rates of net photosynthesis (*P*_net_) at 32°C was also observed (Fig. 1B, linear mixed-effects *P* = 0.088), while respiration rates significantly increased (Fig. 1B, linear mixed-effects *P* = 0.001). Overall, these results point to a bleaching process at 32°C. Bulk *δ*^13^C and *δ*^15^N values of the host tissue and symbiont did not show any significant difference between the two temperature conditions of 25°C and 32°C (Fig. 2, linear mixed-effects *P* > 0.25). However, for both temperatures, there was a significant difference in the *δ*^13^C and *δ*^15^N values between the host and symbionts (Fig. 2, linear mixed-effects *P* < 0.001), with higher (more positive) *δ*^13^C values and lower *δ*^15^N values in the symbionts. The host-symbiont difference was larger at 32°C than at 25°C (temperature interaction, Fig. 2B, linear mixed-effects *P* = 0.036).

**Fig 3:**
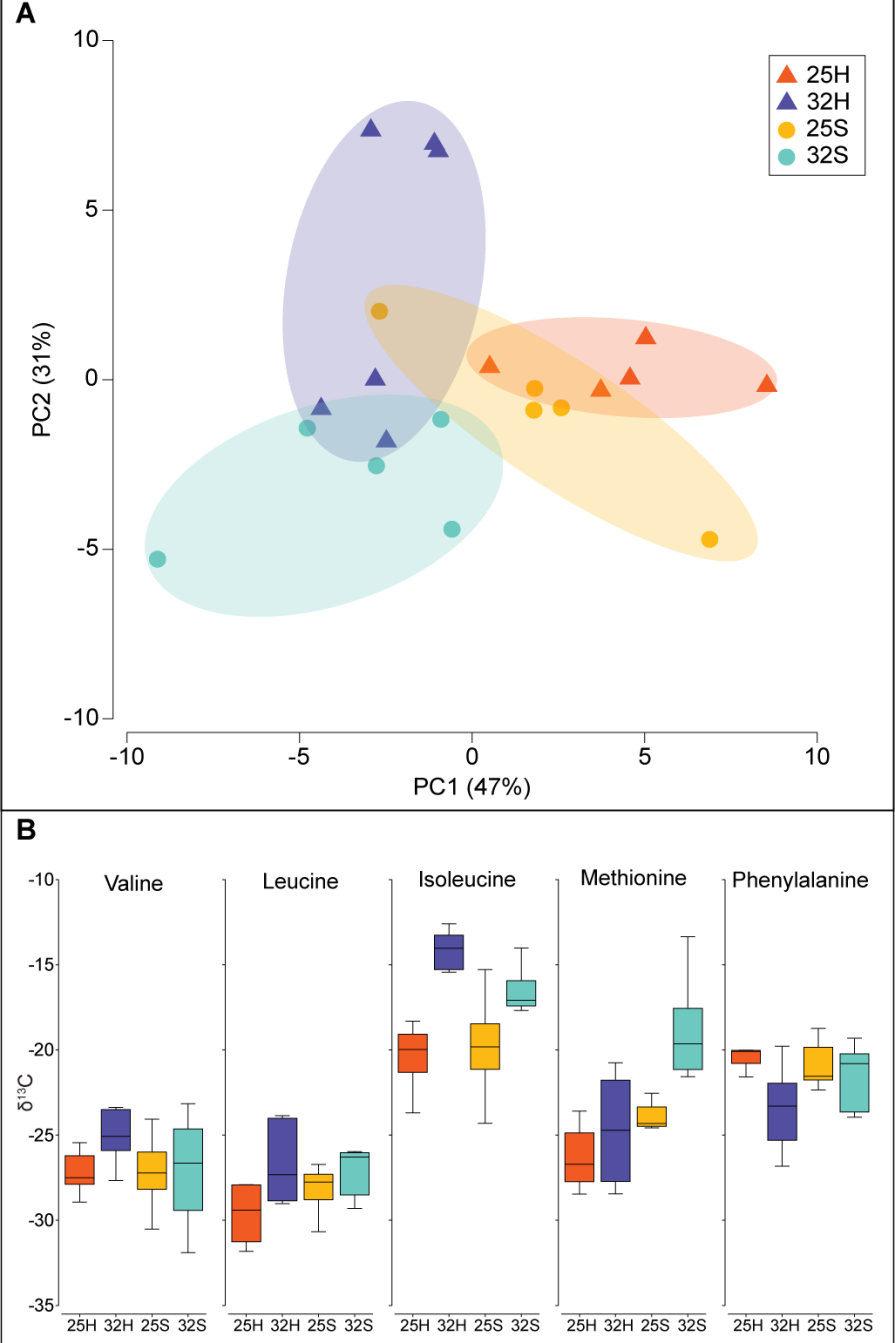
Carbon CSIA-AA of host and symbionts from two temperature treatments of 25°C and 32°C. (**A**) PCA of five essential amino acids (Valine, Leucine, Isoleucine, Methionine, and Phenylalanine). (**B**) Carbon isotopic values of the five essential amino acids. Samples are 25°C host (25H), 32°C host (32H), 25°C symbionts (25S), and 32°C symbionts (32S).

**Fig 4:**
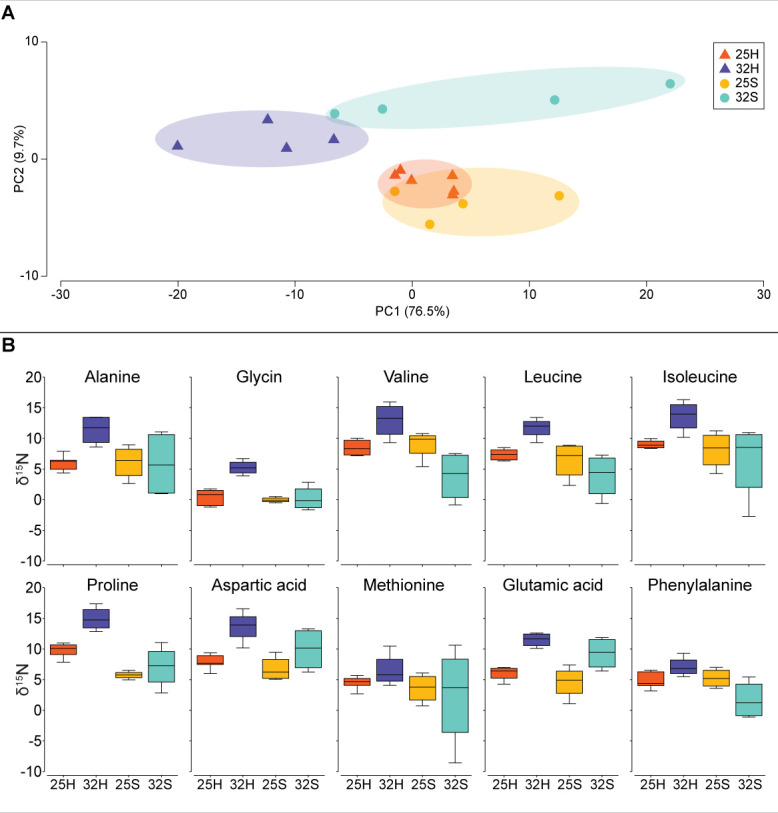
*δ*^15^N-AA values of host and symbionts from two temperature treatments of 25°C and 32°C. (**A**) PCA of 10 amino acids (Alanine, Glycine, Valine, Leucine, Isoleucine, Proline, Aspartic acid, Methionine, Glutamic acid, and Phenylalanine). (**B**) *δ*^15^N-AA values of the 10 amino acids. Samples are 25°C host (25H), 32°C host (32H), 25°C symbionts (25S), and 32°C symbionts (32S).

**Fig 5:**
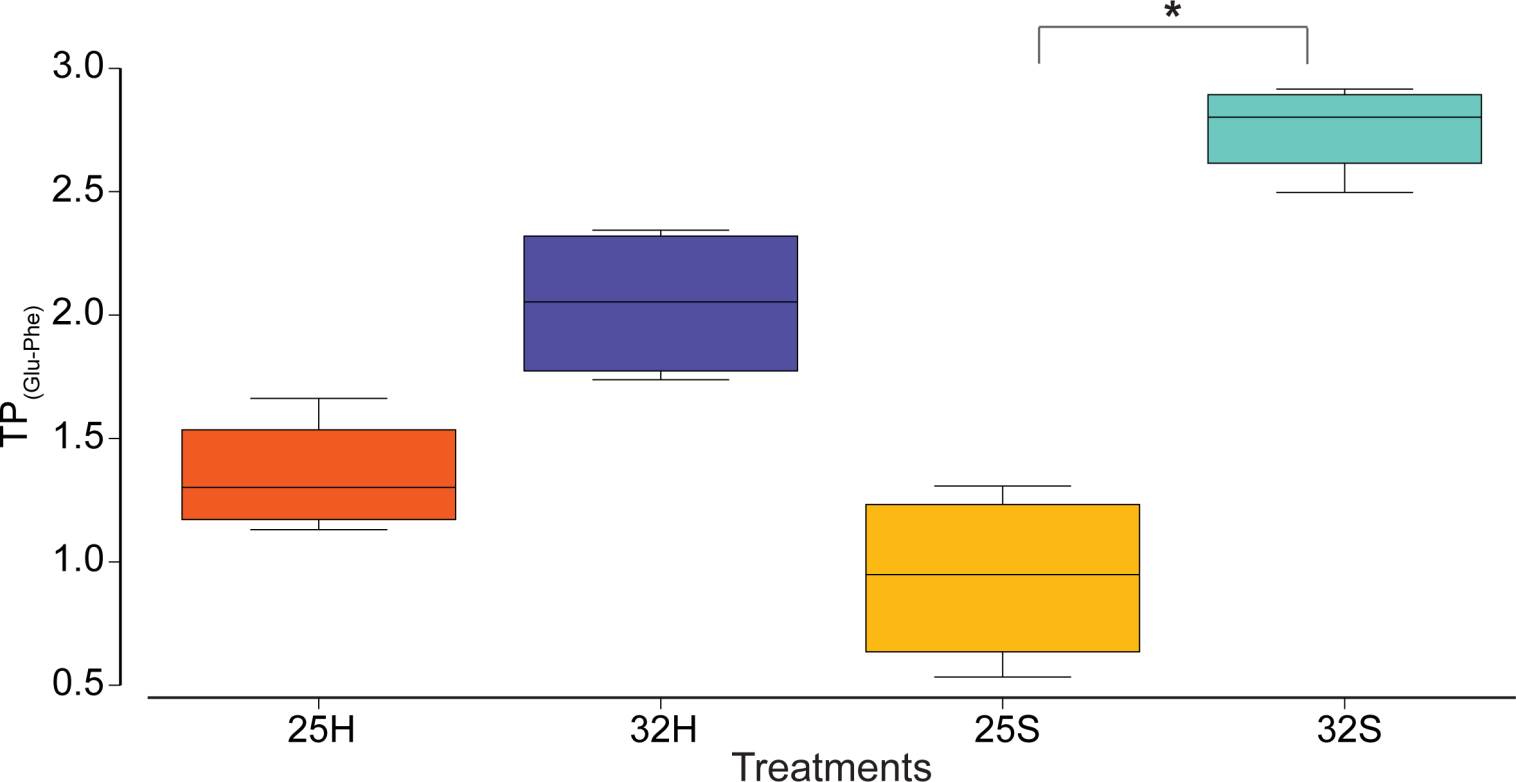
Calculated trophic position (TP_(Glu-Phe)_) from two amino acids (glutamic acid and phenylalanine) of the following samples 25°C host (25H), 32°C host (32H), 25°C symbionts (25S), and 32°C symbionts (32S). * indicates a significant difference.

The *δ*^13^C and *δ*^15^N-AA values of the *Artemia salina* nauplii that were used to feed the corals are presented in Table 1. Methionine had the highest *δ*^13^C value (−20.2‰), while valine had the lowest (−30.3‰). *δ*^15^N values ranged from 3.3‰ for glycine to 17.1‰ for valine. Temperature, host-symbiont fraction, and their interaction had a significant effect on the *δ*^13^C -AA values of the five essential amino acids of the coral tissue (Valine, Leucine, Isoleucine, Methionine, and Phenylalanine) (permanovaFL *P* < 0.001, *P* < 0.001, and *P* < 0.001, respectively, Fig. 3A and B) although no directional effect (increase or decrease) could be detected across the individual AAs (Fig. 3B). The most evident change was for isoleucine, which had higher *δ*^13^C values in the host and symbionts at 32°C, as well as methionine, with higher values in the symbionts at 32°C (Fig. 3B). The PCA however showed a clear separation between the two temperature conditions, for both the host and the symbionts with statistical significance (Fig. 3A, pairwise PERMANOVA MC *P* = 0.004 and *P* = 0.018, respectively), as well as between the host and symbionts at 32°C (Fig. 3A, pairwise PERMANOVA MC *P* = 0.014). Temperature, host-symbiont fraction, and their interaction had a significant effect on the *δ*^15^N-AA values of the coral tissue (permanovaFL *P* = 0.001 and *P* = 0.017, respectively, Fig. 4A and B). The *δ*^15^N-AA values of the host were higher at 32°C compared to 25°C, while the values of the symbionts were not (Fig. 4A, pairwise PERMANOVA MC *P* = 0.01 and *P* = 0.277, respectively). There was a significant difference between the host and symbionts at 32°C, with higher values in the host (Fig. 4A, pairwise PERMANOVA MC *P* = 0.027). The calculated trophic position (TP_(Glu-Phe)_) did not show a significant change between temperature or the host-symbiont fraction (Fig. 5, linear mixed-effects *P* = 0.19 and *P* = 0.12, respectively). However, their interaction between temprature and host-symbiont fraction showed a significant change (linear mixed-effects *P* = 0.003) due to the increase from 0.9 to 2.75 in TP_(Glu-Phe)_ of the symbionts at 32°C.

**TABLE 1:** Isotopic values (‰) of nitrogen and carbon in Artemia salina nauplii. --- indicates missing data

Amino acids	*δ*^15^N	*δ*^13^C
Alanine	16.5	---
Glycine	3.3	---
Valine	17.1	−30.3
Leucine	16.4	−27.7
Isoleucine	15.2	−20.8
Proline	11.2	---
Aspartic acid	12.5	---
Methionine	7.6	−20.2
Glutamic acid	13.3	---
Phenylalanine	8.2	−25.1

**Fig 6:**
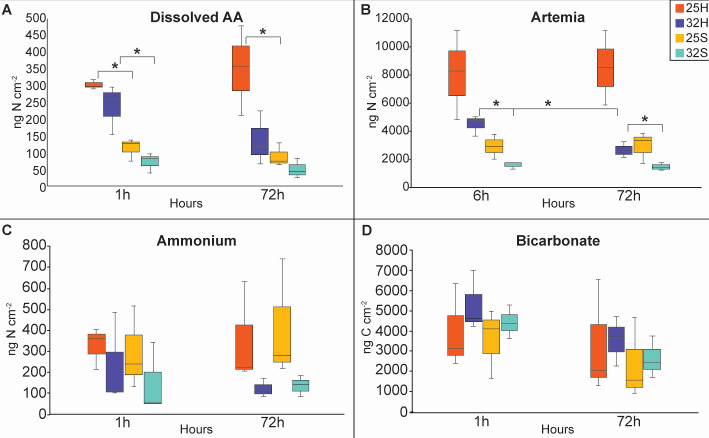
Bulk isotope analysis of isotopically enriched coral hosts and symbionts at two temperatures (25°C and 32°C) sampled at 1 and 72 h after enrichment. (**A**) ^15^N-dissolved amino acids. (**B**) ^15^N-labeled *Artemia salina* nauplii. (**C**) ^15^N-labeled ammonium. (**D**) ^13^C-labeled bicarbonate. 25°C host (25H), 32°C host (32H), 25°C symbionts (25S), and 32°C symbionts (32S). * indicates a significant difference.

After 1 h of incubation with dissolved free amino acids (DFAA), uptake rates were at least twice as high in the host than in the symbionts, both at 25°C and at 32°C (Fig. 6A, pairwise PERMANOVA MC *P* = 0.002 and *P* = 0.031, respectively). This suggests that the host first takes up the DFAAs before passing them on to the symbionts. Additionally, there was no significant difference in uptake rates between the two temperature conditions. After 72 h, the host retained three times more DFAAs than the symbionts at 25°C (pairwise PERMANOVA MC *P* = 0.026), but not at 32°C. Six hours after incubation with labeled *A. salina*, assimilation of nitrogen was three times higher in the host than in the symbionts at both temperatures, with a significant decrease in assimilation rates measured at 32°C (Fig. 6B, pairwise PERMANOVA MC *P* = 0.005). The treatments with labeled ammonium and bicarbonate (Fig. 6C and D, respectively) indicate that there was no significant difference in uptake rates between temperature conditions, host-symbiont fractions, or time. This suggests that assimilation rates were similar at both temperatures, with a similar distribution between compartments. Additionally, the results suggest that the loss of these compounds for energy production was low.

## DISCUSSION

Under normal growth conditions of 25°C, all proxies showed strong agreement that *S. pistillata* relied on a mixotrophic diet for its growth. Unlike autotrophic corals, there was no overlap in bulk *δ*^13^C and *δ*^15^N values between the host tissue and the symbionts (14). The *δ*^13^C values of *S. pistillata* were also significantly lower than those typically found in purely autotrophic coral holobionts (14, 33). However, the particularly low *δ*^13^C values measured in this experiment suggest that factors other than heterotrophy, such as low light levels or internal recycling, may have influenced the *δ*^13^C value (34, 35). The trophic position (TP_(Glu-Phe)_), which was 1.3 for the host, confirmed the mixotrophic diet of *S. pistillata* at 25°C, while the symbionts had a TP_(Glu-Phe)_ of almost 1, indicating autotrophy (25, 26). Furthermore, *S. pistillata* was able to feed on both DFAAs and *A. salina* nauplii. Nitrogen assimilation rates from these organic sources were higher in the host than in the symbionts, consistent with previous observations (36, 37). Nevertheless, the symbionts rapidly assimilated ca. 30% of the nitrogen from these heterotrophic food sources, either from the coelenteric digestion of the prey or from translocation (38, 39). Importantly, nitrogen was retained within the symbiotic association, as no significant decrease was observed after 72 h, suggesting that the heterotrophically acquired nitrogen contributed to host tissue buildup (26, 40). Nitrogen from ammonium uptake was also retained in the symbiotic association but equally assimilated in the host and symbiont biomass, as both fractions have the ability to fix ammonium (5, 41).

Although the holobiont had a mixotrophic diet, the CSIA-AA results measured at 25°C showed a similarity in the *δ*^13^C and *δ*^15^N-AAs of the host and symbionts (Fig. 3 and 4). This suggests that under normal growth conditions, the coral holobiont relied on autotrophy for amino acid synthesis (14). These observations are confirmed by the significantly lower *δ*^15^N values of glutamic acid, the main trophic amino acid, in the coral tissue (5‰–6‰) compared to the artemia prey (13.3‰). A similar pattern is observed with the source amino acid phenylalanine, which had lower values in the coral (5‰–6‰) than in the artemia (8.2‰). In addition, the higher *δ*^13^C value of isoleucine relative to other AAs (25) indicates a high contribution of dissolved inorganic carbon (DIC) to isoleucine biosynthesis. This is consistent with the fact that DIC in seawater typically has *δ*^13^C values ranging from −0.5% to 1.8‰ (42) which are much higher than those of organic matter. This further supports the reliance of the coral holobiont on autotrophic processes for amino acid synthesis under these conditions.

Thermal stress resulted in significant bleaching in *S. pistillata*, characterized by a significant decrease in symbiont density and rates of net photosynthesis by ca. 6- and 2-fold, respectively, at 32°C compared to 25°C. However, there was no change in the assimilation of DIC or ammonia through photosynthesis. Altogether, these results suggest that the autotrophic capacity of *S. pistillata* remained optimal at high temperatures and that the symbionts captured light more efficiently at low than at high densities. This is consistent with previous studies, showing that *S. pistillata* is a thermotolerant species that does not lose its photosynthetic capacity at temperatures below 33°C (43, 44). Furthermore, the bulk *δ*^13^C and *δ*^15^N values of both the host and symbionts did not change significantly between 25°C and 32°C, indicating sustained autotrophic function. However, the *δ*^15^N values exhibited a greater difference between the host and symbionts at 32°C than at 25°C, suggesting that the host started to rely on another nitrogen source compared to the symbionts, likely transitioning from an autotrophic to a heterotrophic source. A longer incubation period at 32°C would likely have resulted in a significant change in the bulk *δ*^15^N values of the host tissue (45).

The shift toward higher heterotrophy of *S. pistillata* at 32°C was further confirmed by the results of *δ*^13^C-AAs values, which revealed a change in the symbiotic relationship between host and symbionts in heat-stressed corals. The *δ*^13^C-AAs and the *δ*^15^N-AAs values of heat-stressed corals, which overlapped in the PCA between the host and symbionts at 25°C, no longer overlapped at 32°C, suggesting different carbon and nitrogen sources between the two partners for their amino acid acquisition. In particular, there was a significant increase in the *δ*^13^C value of methionine for the symbionts, and a significant increase in the *δ*^13^C-isoleucine of the host and symbionts at 32°C compared to 25°C. In agreement with the *δ*^13^C-AAs values, the significant increase in the *δ*^15^N values of all amino acids of the host at 32°C toward the values of the *A. salina* prey (25) indicates that the host relied on heterotrophy for its acquisition of amino acids. The increase in the *δ*^15^N values of glutamic acid of the symbionts at 32°C also shows that they acquired the nitrogen required for this synthesis through heterotrophy. The glutamic acid, as a precursor, was then possibly used by the symbionts to synthesize amino acids.

Finally, a higher reliance on heterotrophy at 32°C was confirmed by the significant increase in the TP_(Glu-Phe)_ from 0.9 to 2.75 for the symbionts and an increase from 1.35 to 2.05 for the host, values characteristic of almost completely heterotrophic organisms. As previously observed, the transition from autotrophy to heterotrophy was more pronounced in the TP_(Glu-Phe)_ of the symbionts than in the host (25, 26). This could be due to the slower turnover times of the host compared to the symbionts. The high trophic position observed in symbionts at 32°C seems to be in contradiction to their low nitrogen acquisition rates from labeled *A. salina*. However, this can be explained by the fact that the biomass of symbionts is six times smaller than that of the host. Since the biomass of the symbiont compartment is taken into account when calculating nitrogen uptake rates, a moderate nitrogen flux can still lead to a high *δ*^15^N enrichment. Moreover, the significant increase in the trophic position of the heat-stressed symbionts was a result of both an increase in the *δ*^15^N value of glutamic acid and a decrease in the *δ*^15^N value of phenylalanine. This latter usually remains unchanged when transferred between different trophic positions (24) but can be altered when the nitrogen source switches from a dissolved form such as ammonium or nitrate to a particulate form such as *A. salina* prey. As a result, the trophic index of 2.75 for the symbionts can be slightly overestimated since the coral diet has not yet reached a steady state and is still changing. Lastly, the results of the experiment with ^15^N-labeled artemia or ^15^N-labeled amino acids show that at 32°C, the ^15^N labeling decreased in the host tissue after 72 h, suggesting that the heterotrophically acquired nitrogen was used for energy purposes (26, 40). The decrease in retained nitrogen at 32°C after 72 h may also indicate higher metabolism at this temperature and greater energy demand, inducing a shorter turnover time of the cells.

In summary, this study underscores the importance of using multiple indicators to gain a comprehensive understanding of coral nutrition and the changes in diet in response to thermal stress. The results obtained with the different isotopic markers suggest that although heat-stressed *S. pistillata* decreased its grazing rates on plankton, heterotrophy was still essential for amino acid acquisition by the host and synthesis by the symbionts. These results highlight that a coral’s reliance on heterotrophy is not simply a function of the amount of prey consumed or a reduction in autotrophic vs heterotrophic availability. They indicate that carbon and nitrogen for amino acid synthesis came from autotrophic sources under normal conditions and from heterotrophic sources under heat stress conditions. Consequently, *S. pistillata* shows an adaptive ability to use its food sources in different ways depending on the prevailing environmental conditions.

## Data Availability

All data generated or analyzed during this study are included in this published article.

## SUPPLEMENTAL MATERIAL

Table S1 mbio.01966-24-s0001.docx Statistical results.

ASM does not own the copyrights to Supplemental Material that may be linked to, or accessed through, an article. The authors have granted ASM a non-exclusive, world-wide license to publish the Supplemental Material files. Please contact the corresponding author directly for reuse.
