# Supplementary material for: Unveiling the importance of heterotrophy for coral symbiosis under heat stress
Source: mBio. 2024 Aug 29;15(10):e01966-24. doi: 10.1128/mbio.01966-24 (PMC11481558; doi:10.1128/mbio.01966-24)
Supplement: Table S1 — Statistical results. [file mbio.01966-24-s0001.docx]

Supplementary table 1: Results of statistical analyses. Significant p-values between 0.05-0.01 are marked with *, 0.01-0.001 with **, and p<0.001 ***.

|  | Test | t | df | p-value | Significance |
| --- | --- | --- | --- | --- | --- |
| **Physiology** |  |  |  |  |  |
| Symbiont density | linear mixed-effects | -8.4849 | 4.154e+35 | <0.001 | *** |
| photosynthesis light | linear mixed-effects | 1.89071 | 1.890711 | 0.088 |  |
| photosynthesis dark | linear mixed-effects | 4.25202 | 10 | 0.001 | *** |
| **Bulk Carbon** |  |  |  |  |  |
| Treatment | linear mixed-effects |  | 14.999997 | 0.262 |  |
| Host/symbiont | linear mixed-effects |  | 14.999997 | 0.001 | *** |
| Treatment: Host/symbiont | linear mixed-effects |  | 14.999997 | 0.670 |  |
| **Bulk Nitrogen** |  |  |  |  |  |
| Treatment | linear mixed-effects |  | 20 | 0.252 |  |
| Host/symbiont | linear mixed-effects |  | 20 | <0.001 | *** |
| Treatment: Host/symbiont | linear mixed-effects |  | 20 | 0.03 | * |
| **CSIA-AA Carbon** |  |  |  |  |  |
| Treatment | permanovaFL |  | inf | <0.001 | *** |
| Host/symbiont | permanovaFL |  | inf | <0.001 | *** |
| Treatment: Host/symbiont | permanovaFL |  | inf | <0.001 | *** |
| Host 25:32 | PERMANOVA MC | 2.8356 |  | 0.004 | ** |
| Symbiont 25:32 | PERMANOVA MC | 1.9088 |  | 0.018 | ** |
| 32 host:symbiont | PERMANOVA MC | 2.0885 |  | 0.014 | * |
| 25 host:symbiont | PERMANOVA MC | 1.1217 |  | 0.308 |  |
| **CSIA-AA Nitrogen** |  |  |  |  |  |
| Treatment | permanovaFL |  | 0.4091869 | 0.664 |  |
| Host/symbiont | permanovaFL |  | 5.8097721 | 0.001 | *** |
| Treatment: Host/symbiont | permanovaFL |  | 2.7722583 | 0.017 | * |
| Host 25:32 | PERMANOVA MC | 4.4047 |  | 0.001 | *** |
| Symbiont 25:32 | PERMANOVA MC | 1.134 |  | 0.296 |  |
| 32 host:symbiont | PERMANOVA MC | 2.4144 |  | 0.029 | * |
| 25 host:symbiont | PERMANOVA MC | 1.4167 |  | 0.146 |  |
| **Trophic position** |  |  |  |  |  |
| Treatment | linear mixed-effects | 1.35405 | 12.443901 | 0.199 |  |
| Host/symbiont | linear mixed-effects | -1.6649 | 12.443901 | 0.120 |  |
| Treatment: Host/symbiont | linear mixed-effects | 3.69756 | 11.92081 | 0.003 | ** |
| **Dissolved free amino acids** |  |  |  |  |  |
| Host 25 1:72h | PERMANOVA MC | 0.61069 |  | 0.576 |  |
| Host 32 1:72h | PERMANOVA MC | 1.5886 |  | 0.179 |  |
| Symbiont 25 1:72h | PERMANOVA MC | 0.89846 |  | 0.422 |  |
| Symbiont 32 1:72h | PERMANOVA MC | 0.8903 |  | 0.440 |  |
| 1h 25-host:32-host | PERMANOVA MC | 1.4972 |  | 0.174 |  |
| 1h 25-host:25-symbiont | PERMANOVA MC | 8.9853 |  | 0.002 | ** |
| 1h 25-symbiont:32-symbiont | PERMANOVA MC | 1.5891 |  | 0.182 |  |
| 1h 32-host:32-symbiont | PERMANOVA MC | 3.5772 |  | 0.031 | * |
| 72h 25-host:32-host | PERMANOVA MC | 2.3568 |  | 0.082 |  |
| 72h 25-host:25-symbiont | PERMANOVA MC | 3.265 |  | 0.026 | * |
| 72h 25-symbiont:32-symbiont | PERMANOVA MC | 1.4432 |  | 0.234 |  |
| 72h 32-host:32-symbiont | PERMANOVA MC | 1.7261 |  | 0.151 |  |
| ***Artemia salina*** |  |  |  |  |  |
| Host 25 6:72h | PERMANOVA MC | 0.14439 |  | 0.890 |  |
| Host 32 6:72h | PERMANOVA MC | 3.4356 |  | 0.024 | * |
| Symbiont 25 6:72h | PERMANOVA MC | 0.03975 |  | 0.977 |  |
| Symbiont 32 6:72h | PERMANOVA MC | 0.5346 |  | 0.651 |  |
| 1h 25-host:32-host | PERMANOVA MC | 1.9116 |  | 0.123 |  |
| 1h 25-host:25-symbiont | PERMANOVA MC | 2.7024 |  | 0.053 |  |
| 1h 25-symbiont:32-symbiont | PERMANOVA MC | 2.476 |  | 0.062 |  |
| 1h 32-host:32-symbiont | PERMANOVA MC | 6.5367 |  | 0.005 | ** |
| 72h 25-host:32-host | PERMANOVA MC | 2.9192 |  | 0.058 |  |
| 72h 25-host:25-symbiont | PERMANOVA MC | 2.6066 |  | 0.086 |  |
| 72h 25-symbiont:32-symbiont | PERMANOVA MC | 2.2127 |  | 0.09 |  |
| 72h 32-host:32-symbiont | PERMANOVA MC | 3.2327 |  | 0.036 | * |
| **Ammonium** |  |  |  |  |  |
| Host 25 1:72h | PERMANOVA MC | 0.18123 |  | 0.862 |  |
| Host 32 1:72h | PERMANOVA MC | 0.86395 |  | 0.451 |  |
| Symbiont 25 1:72h | PERMANOVA MC | 0.5776 |  | 0.597 |  |
| Symbiont 32 1:72h | PERMANOVA MC | 0.12449 |  | 0.896 |  |
| 1h 25-host:32-host | PERMANOVA MC | 0.68658 |  | 0.552 |  |
| 1h 25-host:25-symbiont | PERMANOVA MC | 0.23149 |  | 0.832 |  |
| 1h 25-symbiont:32-symbiont | PERMANOVA MC | 0.97697 |  | 0.370 |  |
| 1h 32-host:32-symbiont | PERMANOVA MC | 0.5166 |  | 0.644 |  |
| 72h 25-host:32-host | PERMANOVA MC | 1.6471 |  | 0.176 |  |
| 72h 25-host:25-symbiont | PERMANOVA MC | 0.27349 |  | 0.808 |  |
| 72h 25-symbiont:32-symbiont | PERMANOVA MC | 1.6573 |  | 0.710 |  |
| 72h 32-host:32-symbiont | PERMANOVA MC | 0.41718 |  | 0.147 |  |
| **Bicarbonate** |  |  |  |  |  |
| Host 25 1:72h | PERMANOVA MC | 0.32345 |  | 0.777 |  |
| Host 32 1:72h | PERMANOVA MC | 1.5497 |  | 0.193 |  |
| Symbiont 25 1:72h | PERMANOVA MC | 0.79468 |  | 0.444 |  |
| Symbiont 32 1:72h | PERMANOVA MC | 2.3292 |  | 0.082 |  |
| 1h 25-host:32-host | PERMANOVA MC | 0.89032 |  | 0.408 |  |
| 1h 25-host:25-symbiont | PERMANOVA MC | 0.24352 |  | 0.811 |  |
| 1h 25-symbiont:32-symbiont | PERMANOVA MC | 0.75972 |  | 0.475 |  |
| 1h 32-host:32-symbiont | PERMANOVA MC | 0.88271 |  | 0.407 |  |
| 72h 25-host:32-host | PERMANOVA MC | 0.14178 |  | 0.892 |  |
| 72h 25-host:25-symbiont | PERMANOVA MC | 0.46485 |  | 0.657 |  |
| 72h 25-symbiont:32-symbiont | PERMANOVA MC | 0.20274 |  | 0.405 |  |
| 72h 32-host:32-symbiont | PERMANOVA MC | 0.99089 |  | 0.472 |  |
